# Epilepsy, Regulation of Brain Energy Metabolism and Neurotransmission

**DOI:** 10.2174/092986709787549316

**Published:** 2009-03

**Authors:** Jean-François Cloix, Tobias Hévor

**Affiliations:** Laboratoire de Neurobiologie, Université d’Orléans, BP 6759, 45067 Orléans Cedex 2, France

**Keywords:** Epilepsy, methionine sulfoximine, chemically-induced epilepsy, glycogen, astrocytes, glycogenesis, gluconeogenesis.

## Abstract

Seizures are the result of a sudden and temporary synchronization of neuronal activity, the reason for which is not clearly understood. Astrocytes participate in the control of neurotransmitter storage and neurotransmission efficacy. They provide fuel to neurons, which need a high level of energy to sustain normal and pathological neuronal activities, such as during epilepsy. Various genetic or induced animal models have been developed and used to study epileptogenic mechanisms. Methionine sulfoximine induces both seizures and the accumulation of brain glycogen, which might be considered as a putative energy store to neurons in various animals. Animals subjected to methionine sulfoximine develop seizures similar to the most striking form of human epilepsy, with a long pre-convulsive period of several hours, a long convulsive period during up to 48 hours and a post convulsive period during which they recover normal behavior. The accumulation of brain glycogen has been demonstrated in both the cortex and cerebellum as early as the pre-convulsive period, indicating that this accumulation is not a consequence of seizures. The accumulation results from an activation of gluconeogenesis specifically localized to astrocytes, both *in vivo *and *in vitro*. Both seizures and brain glycogen accumulation vary when using different inbred strains of mice. C57BL/6J is the most “resistant” strain to methionine sulfoximine, while CBA/J is the most “sensitive” one. The present review describes the data obtained on methionine sulfoximine dependent seizures and brain glycogen in the light of neurotransmission, highlighting the relevance of brain glycogen content in epilepsies.

## 1. INTRODUCTION

Glutamate and γ-aminobutyric acid (GABA) are the most abundant neurotransmitters in the central nervous system (CNS). The action potential transmission operated by the excitatory amino-acid glutamate is mediated through two kinds of receptors: (i) ionotropic receptors and (ii) metabotropic receptors. Three classes of ionotropic receptors to glutamate are commonly described, i.e. AMPA (α-amino-3-hydroxy-5-methylisoazol-4-propionate), NMDA (N-methyl-D-aspartate) and kainate (kainic acid) receptors. The activation of these receptors induces a K^+^ ion outward and a Na^+^ ion inward transport, which in turn induces activation of action potential. Two types of the GABA receptors have been described: GABA_A_ and GABA_B_. The activation of GABA_A_ receptors induces an inward entry of chloride ions, while that of GABA_B_ receptors induces an outflow of K^+^ ions, both actions lead to an inhibition of neuronal activity. These two complementary and contradictory neurotransmission pathways constitute the targets for most antiepileptic drugs. Besides neurons, glial cells constitute the majority of brain cells and are of four types: (i) ependymocytes, (ii) microgliocytes, (iii) oligodendrocytes and (iv) astrocytes. Astrocytes are the more abundant glial cells, and they have numerous functions including their contribution to neurogenesis [1-3] and synaptogenesis [3-8]. Moreover, the end-feet of astrocytes are also involved in various functions.

Epilepsy is characterized by many symptoms which are manifestations of the various clinical forms of the condition. The World Health Organization (WHO) recognizes at least 40 forms [9-14], which correspond to a sudden and temporary synchronization of neuron activities whose origins are not well understood [15-20]. The balance between excitatory and inhibitory neurons, basically the equilibrium between glutamatergic and GABAergic neurons, controls temporary neuron synchronization. The knowledge of this disequilibrium is one of the keys to a cure for epilepsies. Searches for epileptic mechanisms are based upon clinical approaches, and the need to know where the epileptic foci are localized. Such localization depends on both EEG and medical imageries. The latter are based upon various mechanisms, and the uptake of glucose analogs represents one of them [21-23]. As humans cannot constitute an adequate experimental model for studying the mechanisms responsible for seizures various animal models have been developed and used for this purpose.

The present review briefly describes human epilepsies and animal models developed to study this disease, as well as data obtained for methionine sulfoximine (MSO) dependent seizures and brain glycogen accumulation. The review highlights the relevance of brain glycogen content in epilepsies. Brain glycogen will be determined *in vivo* in the near future, either in animals or in humans, and might therefore be used as a diagnostic tool as well as a therapeutic target.

## 2. EPILEPSY IN HUMAN AND ANIMALS

Epilepsy is a neurological disorder characterized by sudden and temporary bursts of a group of neurons for focal epilepsy, or of “whole” cortex when the crisis is generalized. When febrile epilepsies are excluded, epilepsy affects up to 1% of the population [24]. The most striking form of human epilepsy, the former so-called “grand-mal”, has enormous consequences. It can lead to a difficult life in affected patients, some of who may die from status epilepticus. The reasons why a group of neurons suddenly and temporarily synchronize their activity are not well understood, and need clarification. A better understanding of the condition will provide neuroscientists and physicians with crucial knowledge, which will enable the development of new therapeutic approaches with benefits to patients. Such advances are being based on studies of specific forms of human epilepsies; and although research on human epilepsies is limited, for deontological reasons, it is needed in order to fit the physiological conditions of this disease. Animal models of experimental epilepsies have therefore been developed and widely used, and are essentially classified into three groups: (i) genetic models, (ii) models in which crisis is induced by external stimuli, such as chemical or physical agents and (iii) models in which the responses to external stimuli depend on the genetic background.

Animal models mimicking human epilepsies are peculiar, and each animal model most likely concerns a specific aspect, or a mixture of some aspects of the variety of human epilepsies. Nonetheless these models have high utility and are an important prerequisite for research into epilepsy, as they allow us to approach specific point(s) of the human pathology experimentally. They are also powerful, making it possible to pinpoint a specific aspect of epilepsy and to analyze it in a “simpler” context than in human pathology. 

## 3. EPILEPTOGENESIS

The understanding of the process by which a normal brain becomes epileptic, epileptogenesis, may help to identify molecular targets for drugs that could prevent epilepsy, as the current pharmacological therapy is only symptomatic and never cures the illness. The basic structure and functioning of the neuronal network is well understood, and below we take the example of the network of the cerebral cortex.

### 3.1. EEG Formation

The pyramidal cells are the major projection neurons of the cerebral cortex, and they project their axons into other areas of the brain and the spinal cord. They are excitatory neurons and principally use glutamate as a neurotransmitter. These cells exhibit a large number of collaterals that spread inside the cerebral cortex, and their dendrites often cross several layers of the cerebral cortex and are oriented perpendicular to the surface of the brain. A second kind of cell in the cerebral cortex is the interneural stellate cells that spread their axons vertically in the plane of the cortical column. Stellate cells get action potentials from the thalamus, and convey them to other interneurons or to pyramidal cells in the same column. A third kind of cerebral cortex cells are the basket cells, that are interneurons, which horizontally oriented their axons toward neuron somas. The latter axons envelop the somas and are able to inhibit them using the neurotransmitter GABA. These preceding three kinds of neurons are the main substratum of EEG genesis through the so-called theory of volume conduction [25-28]. The recorded potential from a large number of neurons is of course the sum of the electric activity of individual neurons. The excitatory post-synaptic potential of an individual neuron is the result of an inward current, and it spreads as an extracellular voltage outside the neuron membrane. Its magnitude (microvolt range) is too small to be recorded on the scalp, but when many neurons are taken into account, with large EEG electrodes, such a recording becomes possible. Because of the orientation described above, it is believed that EEG principally comes from pyramidal neurons, since the contribution of the other kinds of cell is almost negligible. It is not easy to distinguish an EEG coming solely from the pyramidal cells, or from a modulation of these pyramidal activities by other neurons, as pyramidal neurons are connected to the thalamus that synchronously activates a large number of these neurons. The main component of EEG is surely extracellular potentials deriving from excitatory post-synaptic potentials, since the latter are slow and can summate [29, 30]. The contribution of action potentials may exist but may be negligible, because its rapidity makes it difficult to summate.

### 3.2. Genesis of Epileptic Waves

It is possible to observe a large amplitude in the EEG in alpha, theta, and delta waves, which is due to a synchronization of the activities of groups of neurons [31-33], after which a normal EEG is observed. In the case of epilepsy a temporary synchronization occurs, although its level is high and it generates large spikes and spikes and waves. Moreover, the synchronization is recurrent. The actual cause of such a synchronization is not well understood [15-20]. In experiments using a topic application of penicillin it was shown that GABA inhibition is blocked. Action potentials are concomitant with the EEG spikes when the individual neuron activities are recorded at the same time. Each neuron makes its own action potential when its threshold of depolarization is reached in a normal brain, but the fundamental question of epileptogenesis is why neighboring neurons all make their action potential at the same time? The current believe of a decrease in inhibition of pyramidal neurons is not convincing. Indeed, if such a decreased inhibition is favorable for an increased firing of a given individual neuron, it does not explain why a group of thousands of neurons make their action potentials simultaneously. Some speculations have been proposed [15-20]. A pyramidal cell does not excite its neighboring pyramidal cell in a normal situation, but in epileptic foci such an excitation may occur [34], although the mechanism of this excitation remains unclear. One possibility is an increase in the extracellular potentials described above. In the epileptic situation, extracellular potential at the level of the soma of a given pyramidal cell could be too high, and it could excite the neighbor pyramidal cells, but this possibility is not tenable without a solid experimental demonstration. The soma of the pyramidal cells is “enveloped” in glial cells, principally in non-myelinating oligodendrocytes and astrocytes, so that the spread of extracellular potentials up to the adjacent pyramidal cells is improbable, unless abnormalities exist in the enveloping glial cells. This observation poses the question of a possible involvement of glial cells in epileptogenesis [35-43].

## 4. NEURAL CELLS, NEUROTRANSMISSION, ENERGY METABOLISM

Glial cells are no longer regarded as the “glue” between neurons, but have many roles necessary to sustain brain functions. In 2003 Ben Barres [44] claimed: “…virtually every aspect of brain development and function involves a neuron-glial partnership. It is no longer acceptable to consider glia as passive support cells.” Indeed, oligodendrocytes and Schwann cells are necessary for high-speed neurotransmission by ensheathing axons and generating nodes of Ranvier. These cells provide insulation and trophic factors to neurons. Moreover, the most abundant glial cells, the astrocytes, have come to be considered as an important partner in neurotransmission, and also in terms of regulators of energy provided to neurons. Many roles in fact are now attributed to astrocytes [44, 45].

### 4.1. Astrocytes as Partners in Neurotransmission

The relevance of glial cells in controlling neurogenesis and synaptogenesis has only recently been acknowledged, presumably because the alteration in the preceding processes during development could explain childhood epilepsies. Furthermore, the end-feet of astrocytes surround the capillaries, and therefore contribute to the maintenance of the BBB [46, 47]. They also ensheath the synaptic cleft, and thus are now considered as the third part of the synapses [44, 48]. The end-feet of astrocytes participate in the control of synaptic neurotransmitters and ion concentrations, such as glutamate and potassium, and therefore of the neurotransmission. Moreover, glial cells, particularly astrocytes, control the fate specification of adult neural stem cells. Indeed, astrocytes from hippocampus are capable of regulating neurogenesis by instructing the stem cells to adopt a neural fate [2]. Differentiation of neuronal stem cells into astrocytes or neurons is highly relevant in maintaining brain function. The question of the issues of neuronal/glial cell identity, and neuronal-glial interactions, in the context of adult neural stem cells biology, and their implication in neurogenesis during development and adulthood, was recently reviewed [49-52].

Astrocytes are also involved in the control of synaptogenesis [4, 6, 53-56]. In the mouse CNS this effect varies according to the brain regions and the type of neurons [57]. Astrocyte-derived cholesterol has been demonstrated to be responsible for an effect of astrocytes on synaptogenesis [3, 58-60]. Astrocyte-derived estrogen increases synaptogenesis through the estrogen receptor-alpha in primary rat cortical neurons in culture [61]. Neurothrophins [62, 63], which are widely expressed in the developing and mature CNS and are well known for their role in promoting neuron survival and differentiation, and thrombospondins [64], which induce ultrastructurally normal synapses that are presynaptically active, are involved in the astrocyte-control of synaptogenesis. The astrocyte-control of synaptogenesis involving numerous mechanisms therefore seems to be more complex than expected, more likely bilateral rather than unilateral.

Astrocytes participate in neurotransmission by regulating concentrations of ions and neurotransmitters in the synaptic cleft, thereby controlling synaptic efficacy [5, 44, 65-67]. The synaptic concentration of neurotransmitters depends primarily upon presynaptic activity. However, the end-feet of astrocytes regulate this concentration, as was unambiguously demonstrated for glutamatergic and GABAergic synapses which are often involved in epilepsy [68, 69]. Astrocytes are responsible for the major part of glutamate uptake after its release from glutamatergic neurons [70]. The processes of astrocytes contain glutamate transporters (GLAST) that are more active than those of neural cells. Glutamate, after entering the astrocytes, is transformed into glutamine by glutamine synthetase (GS), an enzyme that is essentially localized to astrocytes and not to neurons [71]. Glutamine is therefore given to neurons in which it is metabolized either to glutamate or GABA (Fig. (**1**)). Moreover, the K^+^ concentration of the synaptic cleft is also regulated by the end-feet of astrocytes [72-75]. In addition, the active roles of extrasynaptic neurotransmitter receptors and their relevance to neurovascular coupling, and of exocytosis of neurotransmitters from astrocytes, have recently been reviewed [67, 76-78], and the implication of these roles in epilepsy have been evoked [79-81]. Astrocytes contribute to the control of neurotransmission using additional mechanisms: (i) they synthesize a glia-derived soluble acetylcholine-binding protein (AChBP), which is a naturally occurring analog of the ligand-binding domains of the nicotinic acetylcholine receptors (nAChRs) [7]. This lure, or fake, receptor contributes to the regulation of acetylcholine content in the cholinergic synapses, by capturing acetylcholine. (ii) astrocytes produce a protein, the tumor necrosis factor alpha (TNFα), which enhances synaptic efficacy by increasing surface expression of AMPA receptors [8]. All these events may influence neuronal excitability and thus epileptogenesis.

Glial cells, particularly astrocytes, should therefore be regarded as active partners of both the neuron and the synapse in terms of regulators of their synthesis and efficacy. These cells participate in neurotransmission through their effects on neurons and synapses, and through their processes of information *via *the astrocyte network. The complexity of neural information and brain organization is reinforced by new considerations that we have to take into account for the most abundant cells in the CNS, namely glial cells.

### 4.2. Astrocytes as Energy Providers to Neurons

It is now thought that astrocytes also largely contribute to fuel neurons during pathological conditions, such as during periods of intense neural activity when energy demand exceeds the supply of glucose from the blood. This idea might also apply during normal conditions. Indeed, two possibilities might exist to fuel neurons [82, 83]. Firstly, glucose enters astrocytes where it is metabolized down to lactate that is given to neurons to supply their energy demands. This is the astrocytes-neuron lactate shuttle hypothesis (ANLSH) [84-88]. Secondly, glucose enters neurons where it is utilized as the main source of energy. This is the conventional hypothesis.

#### 4.2.1. Glucose Entering the Brain

Glucose is the main source of brain energy. Blood glucose has therefore first to cross the endothelial cell capillaries whose tight junctions constitute the BBB, then the intercellular area and the membranes of end-feet of astrocytes, and lastly the neuron membranes. GLUT proteins deliver glucose from the circulatory system to the brain. The microvascular endothelial cells and astrocytes contain GLUT1, while neurons contain both GLUT1 and GLUT3. Under normal conditions there is tightly limited *in vivo *expression of 45 kDa GLUT1 in neurons [89-92]. GLUT3 was cloned from a variety of mammalian brain cDNA libraries, and in the brain it has been localized almost exclusively to neurons [93-96]. As glucose has been detected in extracellular spaces of the brain [97-101], it is quite possible that neurons are capable of taking it up and metabolizing it. Indeed, Simpson *et al*. [102] calculated that GLUT3 has both a higher affinity for glucose than GLUT1, and at least a fivefold greater transport capacity than GLUT1. They concluded that as there are approximately the same number of GLUT1 on glial and endothelial cells, the combination of lower *K*m and higher capacity enables the neurons preferential access to available glucose. In addition to this glucose uptake by neurons, it may be possible that astrocyte metabolism contributes to feeding neurons according to the ANLSH (Fig. (**1**)). Indeed, it seems likely that glucose enters astrocytes, in which it is metabolized to lactate through the glycolysis pathway, and that astrocytes provide neurons with lactate as fuel [87, 103-113]. Neurons are therefore provided with a metabolite that can be rapidly used by the Krebs cycle (Fig. (**1**)). To fuel neurons, the ANLSH might be directly involved with glucose in parallel with the conventional hypothesis. Indeed, it is highly conceivable that these two hypotheses are not mutually exclusive to each other [114, 115], and the conventional hypothesis would be the prevalent one in the normal state, while the ANLSH might be favored in pathological conditions. Putatively, in pathologies such as epilepsy other forms of abnormal carbohydrate metabolism may also occur (see below).

In addition to glucose transporters the glycolytic product of glucose metabolism, lactate, is transported in and out of neural cells by monocarboxylate transporters (MCT), MCT1 in capillaries [116] and MCT2 in neurons [116-118]. The specific localization of these various transporters is in agreement with the ANLSH between astrocytes and neurons [87, 104-108, 111, 119] as summarized in Fig. (**1**).

#### 4.2.2. Glycogen Synthesis and Degradation

The synthesis of glycogen inside the brain is essentially due to astrocytes [120] and microgliocytes and not to neurons, as the latter do not contain glycogen synthase (GlyS) [103], while the two former cells have this enzyme. Nevertheless neurons, which are capable of gluconeogenesis [121-123], are unable to accumulate glycogen [103, 124-126]. Inside astrocytes, glycogen synthesis might be performed using G-6-P coming either from glucose or from gluconeogenesis. Glycogen is therefore accumulated inside astrocytes [110, 111, 124, 127], and when energy is in high demand by neurons astrocytic glycogen might be metabolized down to lactate [88, 109, 119, 128, 129]. Astrocytic glycogen could therefore be considered as an energy-store for neurons [130-133], in addition to lactate-derived from blood glucose through glycolysis (conventional hypothesis), this latter being possible both in neurons and astrocytes [106, 124, 134-136], although during convulsions in which neurons have a high-energy demand, obtaining glucose *via *the blood stream might not be sufficient. The astrocytic energy-store might therefore be used through glycogen degradation that is performed by glycogen phosphorylase (GP), which is essentially expressed in astrocytes [106, 124]. Moreover, astrocytes contain receptors for glycogenolytic neurotransmitters, such as noradrenaline [108, 137] or vasointestinal peptide [104, 138-140]. K^+^ ions also have an acute glycogenolytic action on astrocytes [128, 141]. *In fine*, the preceding neurotransmitter and ion effects, with their glycogenolytic potencies [105, 109-112, 142, 143], contribute to the production of lactate. Astrocytes therefore represent a source of energy to neurons [144], and they perform this role by various means, such as glycogenolysis and glycolysis.

#### 4.2.3. In Vivo Glycogen as Brain Function Marker

Brain glycogen content is lower than that of liver and muscle. Nevertheless, the glycogen content of the CNS is nowadays also regarded as relevant in brain functions. Glycogen synthesis and breakdown in the brain are beginning to be considered as relevant in normal brain functions, and in brain pathologies. Targeting research on brain glycogen pathways will be promising for both diagnosis and therapy. However, in terms of diagnosis, the *in vivo* measurement of brain glycogen content and synthesis is a challenge for the future, although the use of injection of [1-^13^]C-glucose has made possible the non-invasive measurement of brain glycogen content and turnover in both humans [145, 146] and rats [132, 133, 147-149]. These advances in the determination of brain glycogen will create a new understanding of its functions, roles and metabolism in both normal and in pathological conditions [110, 111, 130, 131, 150]. Once this difficult step has been overcome new tools will emerge to challenge brain glycogen in brain pathologies, and to target it as a treatment.

## 5. MSO-INDUCED EPILEPSY

Among the various epileptic or seizure-induced models, one of them is associated with both crisis and a specific increase in brain glycogen content [124, 125, 151-156]. This model corresponds to seizures induced by a derivative of methionine, namely methionine sulfoximine (MSO).

### 5.1. Discovery of MSO

MSO has been described as the chemical responsible for seizures induced in various animals after consumption of agenized flour, during the mid-1950s [157-159]; and it was therefore used to induce seizures in different animals, essentially rats and mice with characterized EEG alterations before and during seizures [124, 154, 160, 161]. Moreover, some plants of the *Cnestis* genus, from Madagascar and Asia, were used to kill running dogs by causing severe convulsions. The active compound in the seeds of *Cnestis palada* [162], and in the roots of *Cnestis glabra* and *Cnestis polyphylla* [163], was identified as MSO.

### 5.2. MSO as an Epileptogenic Molecule

Animals develop seizures after intraperitoneal administration of MSO, and such seizures mimic the human “grand mal”. They are characterized by disequilibrium, fits, running, myoclony and myotony, with EEG modifications. Animals developed epileptiform seizures 6-8 hours after MSO dosing (pre-convulsive period). The seizures were recurrent over 24-48 hours (convulsive or ictal period), and the animals then recovered normal behavior (post-convulsive period) [124, 151, 164]. During this long pre-convulsive period, the animals present ataxia, akinesia, and equilibrium disorders.

MSO strongly resembles glutamate, and it has been suggested that it can induce seizures by mimicking the effect of this excitatory amino-acid [165, 166]. Indeed, if MSO can replace glutamate in the synaptic cleft, it could stimulate neurotransmission and therefore induce seizures, but this mechanism of action is still not clearly understood. In addition, MSO has also been described as an irreversible inhibitor of GS [166]. Such an inhibition might therefore induce a putative increase of glutamate, which has been suggested as being responsible for the MSO-dependent convulsions [166]. Nonetheless, some reports [167-170] describe a decrease in brain glutamate content after the administration of a convulsive dose of MSO in rats, rather than the expected increase; but chronic inhibition of brain GS by MSO did not induce seizures in mice [171]. The involvement of GS inhibition by MSO in the MSO-dependent convulsions is therefore controversial, and may not even be responsible for MSO-dependent seizures.

## 6. MSO AS A GLYCOGENIC MOLECULE

Searches are based on clinical approaches, with the need to know where the epileptic foci are localized. Such localization depends on both EEG and medical imageries. The latter are based on various mechanisms; the uptake of glucose analogs represents one of them [21-23]. This allowed the demonstration that epileptic foci are hypermetabolic during crisis [23], and hypometabolic during the interictal period [22]. Such a hypometabolism is a reflection of either an intrinsic decrease of cellular glucose uptake, or an intrinsic high level of capacity of brain production of energy, or both. The intrinsic increase in brain energy capacity may be due to various cell alterations, such as glycolysis, gluconeogenesis or glycogenesis; the latter resulting in accumulation of glycogen. Such an increase in glycogen content is difficult to demonstrate in humans, although it has been shown that brain biopsies obtained from the hippocampus of epileptic patients contained high glycogen content in comparison with grey and white matter [172]. Such a high level of glycogen could contribute to high-energy demands by neurons during synchronization of their activities. Indeed, during seizures when neurons need a lot of energy, as they are starved by their high activities, this energy might come from different sources: blood supply, neuron glycolysis and astrocyte source. When the first two sources are depleted the astrocyte glycogen might be mobilized, and astrocyte glycogenolysis could then generate lactate, which could be transferred to the neurons.

In MSO-dependent seizures the accumulation of brain glycogen is observable during the pre-convulsive period before crisis, demonstrating that such an accumulation is not a consequence of seizures [124-126, 151, 164]. This high level of glycogen and its highest augmentation are specifically localized in the cortex and cerebellum, and more precisely in astrocytes (Fig. (**2**)) [124-126]. Moreover, the action of MSO on alterations in metabolism is also demonstrable in cultured rat and mouse astrocytes, again showing that such metabolic effects are not consequences of seizures [124, 151, 155, 164]. Conversely, a decrease of glycogen content is also observed in other models of induced seizures, for example seizures induced by homocysteic acid [173, 174], indoklon/flurothyl [175], PTZ [176], hypoglycemia [177], bicuculine [178, 179], and maximal electroshock [177]. The accumulation of glycogen is essentially due to astrocyte metabolism, although in progressive myoclonus epilepsy inclusion Lafora bodies, resembling abnormal glycogen, accumulate in this disease [180]. In addition, and contrary to previous data, it has been recently reported that mouse neurons have enzymatic machinery for synthesizing glycogen [181]. Nonetheless, the accumulation of glycogen in MSO-dependent seizures is specific to astrocytes, and might be relevant in the onset of seizures and in the maintenance of crisis [151, 164].

## 7. MSO EPILEPTOGENESIS IN RELATION WITH NEUROTRANSMISSION

As the excitability of neurons is largely monitored by neurotransmitters many investigations have been devoted to abnormalities in neurotransmission during epilepsy [182-184]; yet despite a large number of experiments we still have no clear explanation on the involvement of neurotransmission in epilepsy. The general believe is that of a defect in the inhibitory neuronal circuits related to GABA, and/or an enhancement of excitatory circuits related to glutamate. The difficulty with this idea is that many epileptogenic situations do not involve defects in either GABA or in glutamate neurotransmissions. One illustration is the model created by organophosphoric compounds such as soman [185-187]. It is well established that soman principally targets cholinergic neurotransmission, and generates typical epileptic seizures involving EEG and convulsions. Moreover, many other models clearly involve catecholamine and indoleamine neurotransmissions [182-184]. The impairment of different neurotransmission systems is thus surely able to generate a variety of epileptic seizure models in animals, and probably different kinds of epilepsies in human as well. We looked for changes in the content of many neurotransmitters in the brain of rodents developing seizures after an intraperitoneal administration of the drug MSO. The aim was to discover any possible relationship between the impairment in neurotransmission and the impairment in the brain carbohydrate metabolism, and MSO-dependent convulsions. Briefly, MSO induced a decrease in dopamine and serotonin levels and an increase in acetylcholine, while the noradrenaline level showed only a small change [188-191]. Using both agonists and antagonists of these neurotransmitters we were able to modulate the seizures, except in the case of acetylcholine. At the same time MSO induced a large increase in glycogen content in all areas of the brain, as previously described [124-126, 151, 154, 164, 178].

The question now arises as to what neurotransmission impairment has to do with glycogen content in epileptogeny? Experiments *in vivo* or *in vitro* [192, 193], and our unpublished data, show that the glycogen content in the brain is insensitive to changes in acetylcholine. So the involvement of a cholinergic system in glycogen accumulation induced by MSO in the brain is improbable. GABA may not cause MSO epileptic-like activity in rodents, nor a glycogen level increase, because the level of this neurotransmitter is insensitive to the convulsant [188]. Biogenic amines, such as catecholamines, indolamines and histamine, have been reported to elicit glycogenolysis in the brains of various species, including mammals [194-197]. As glycogen accumulation is restricted to astrocytes in the brain and neurotransmitter release to neurons, it is obvious that interactions between neurons and astrocytes are responsible for changes in the metabolism of glycogen and of catecholamines and indoleamines. In the case of MSO, the observed decrease in dopamine and in serotonin levels is in a good agreement with the increase in glycogen content in the brain. Does the neurotransmitter decrease therefore trigger the glycogen accumulation, or does glycogen accumulation induce changes in the preceding biogenic neurotransmission system? At present our experimental data does not allow us to clearly identify the primer of change. One can speculate that MSO decreases dopamine and serotonin levels, and thus causes an imbalance between GlyS and GP activities, and that this change results in the glycogen level increase. We have also speculated on the possible sequestration of glucose by astrocytes under the effect of MSO. Primarily, astrocytes may incorporate molecules of glucose into glycogen. Moreover, over the same period of time, *via *an enhanced gluconeogenesis, lactate and pyruvate that astrocytes can normally send to neurons as fuel are converted to G-1-P through gluconeogenesis pathway, which is polymerized as glycogen. Gluconeogenesis and glycogen accumulation in astrocytes deprive neurons of energy, and this may alter neuronal function which then results in epileptic-like activity [125].. We now need to find a robust experimental design that will able us to discriminate an epileptogenesis event primarily triggered by changes in glycogen content, or by changes in neurotransmission under the effect of MSO.

## 8. RELATIONSHIP BETWEEN MSO EFFECTS

After MSO administration glycogen content remains high during the convulsive period, and up to the post-convulsive period. We have demonstrated that both seizure latency and glycogen accumulation are dependent on genotype in mice [151, 164]. Using various inbred strains of mice [151, 164], we observe that the latency to MSO-dependent seizures varies (Fig. (**3**)). The strain C57BL/6J presents the longest latency (13.03 ± 0.45 h, n=92), and the lowest susceptibility toward MSO (ED_50_=60 mg/kg), while CBA/J presents the fastest latency (5.95 ± 0.06 h, n=63) and the highest susceptibility toward MSO (ED_50_=30 mg/kg). The CBA/J strain is also unable to accumulate glycogen inside the brain during the preconvulsive period, as opposed to C57BL/6J that does. This accumulation of brain glycogen is specifically localized to astrocytes, as previously described [124-126]. Indeed, MSO induces an activation of gluconeogenesis by a specific increase in activity of the last irreversible gluconeogenic enzyme, fructose-1,6-bisphosphatase (FBPase). Increases in both FBPase and glycogen deposits are specifically localized to astrocytes [124-126, 155, 191]. In addition, primary cultures of astrocytes from these two strains demonstrate that MSO induces accumulation of glycogen only in cultured astrocytes from C57BL/6L, which is mediated by the specific activation of gluconeogenesis [151].

The question of a causal link between glycogen accumulation and seizures remains open. We have demonstrated that at the onset of MSO-dependent seizures the glycogen that was accumulated in cortices before convulsions returns to the basal level (Fig. (**3**)). Such a normalization of glycogen content occurred only in C57BL/6J mice and not in CBA/J ones [151]. These data suggested (i) a mobilization of the high level of brain glycogen at the onset of seizures, which is observed in mice having a long latency to MSO-dependent convulsions, and (ii) that the accumulated glycogen in brain might be used as energy supply when neurons are in a convulsive state. We therefore hypothesized that these two MSO effects, i.e. the elevation of glycogen and its mobilization during seizures, might constitute a way to postpone seizures [151, 164]. Bi-directional selection of MSO sensitive and MSO resistant mice is under investigation, in order to verify such a hypothesis. Preliminary results suggest that glycogen can accumulate after MSO dosing to a greater extent in cortices of MSO resistant mice than in MSO sensitive ones (unpublished data), confirming our hypothesis that high levels of glycogen could delay MSO-induced seizures [151, 164].

## 9. CONCLUSIONS: POTENTIAL OF MSO-DEPENDENT SEIZURES MODEL

The MSO-dependent seizures model presents some advantages compared with other chemically-induced seizures. Our two most important concerns are as follows. (i) The existence of a long preconvulsive period during which it is possible to easily observe changes before the onset of seizures might not be a consequence of crisis. (ii) This model associates an accumulation of brain glycogen with induction of seizures, and therefore allows the analyses of the relationship between seizures and brain glycogen content. This consideration is relevant, as high brain glycogen content has sideration is relevant, as high brain glycogen content has been described in human epilepsy [172]. Moreover, in MSO dependent seizures glycogen accumulates in the brain before the onset of seizures, and remains high during the convulsive period. Nevertheless, such a high brain glycogen content is mobilized at the onset of seizures and returns to high level thereafter. We therefore propose, that such an elevation of brain glycogen could contribute to the postponement of seizures [151, 164], by mechanisms such as astrocyte glycogen mobilization, which provides neuron with energy. In the very near future brain glycogen will be regarded as a diagnostic tool, as well as a therapeutic target [144]. Furthermore, *in vivo* MRI analyses have been performed in various animal models of epilepsy demonstrating that such a tool is valuable and informative, and might provide a clarification of the mechanisms involved in epilepsy [198, 199].

## 10. CONCLUDING REMARKS

Knowing all the mechanisms and biological events responsible for the various forms of human epilepsies still has a long way to go. Nevertheless, the numerous researches performed using human approaches present a doorway to the subject, which is now fully open due to the experimental models developed to study this neuropathology. Even if much is still unknown, new developments are on the way to giving us a deeper insight into the underlying mechanisms. For example, the use of transgenic mice could provide us with a useful tool for the genetic “dissection” of various aspects of the pathology, and may eventually lead to a “full” understanding of epilepsies. In any event such goals depend upon a better knowledge of brain physiology, which in itself needs the development of new approaches, technologies and ideas. Concerning this new knowledge, the brain glycogen content might be considered as highly relevant, and its *in vivo* measurement of content and variation in humans during normal and pathological conditions will be soon possible [145, 146]. Such a determination will be highly useful for analyzing the potency and relevance of brain glycogen content, and its mobilization in patients during pathological conditions.

## Figures and Tables

**Fig. (1):**
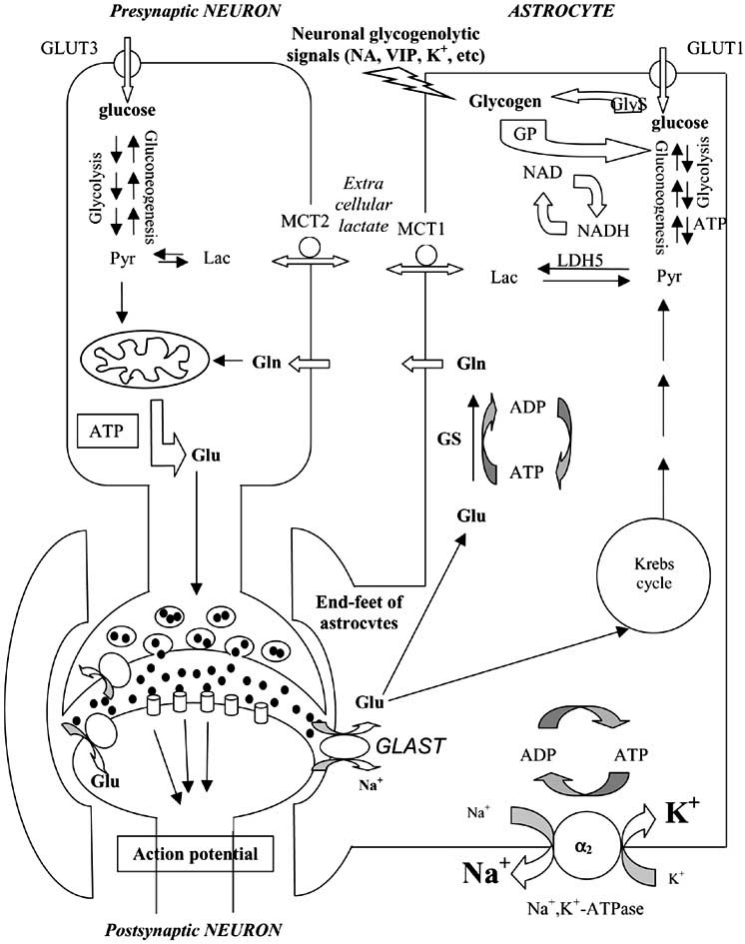
Schematic representation of the glucose-lactate shuttle between neurons and astrocytes (adapted from Pellerin [119], and Cloix [144]). Gln: glutamine, GlyS: glycogen synthase, GP: glycogen phosphorylase, GS: glutamine synthetase, GLAST, GLT-1: Na^+^-glutamate co-transporter, Glu: glutamate, Lac: lactate, LDH5: Lactate dehydrogenase 5, MCT: monocarbohydrate transporter, NA: noradrenaline, Pyr: pyruvate, VIP: vasointestinal peptide.

**Fig. (2):**
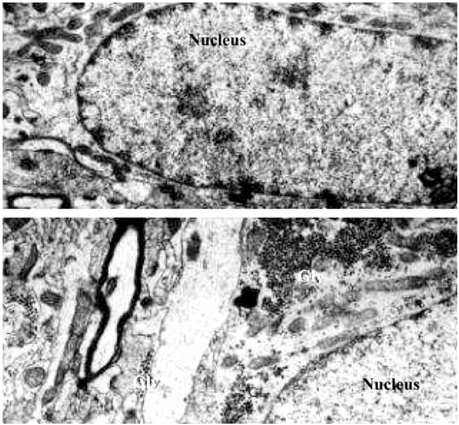
Electron micrographs of sections made in the parietal cerebral cortex of mice after the administration of methionine sulfoximine. Swiss mice were intraperitoneally injected with 100 mg/kg of methionine sulfoximine (**B**) or with saline as control (**A**). After intracardiac perfusion of glutaraldehyde, the brain tissue were taken off, washed in buffered sucrose, post-fixed in osmium tetroxide, dehydrated in graded alcohol and propylene oxide, and embedded in Effapoxy. The sections were made and stained with uranyl acetate and lead citrate and then, examined in an electron microscope. In control, beta glycogen particles are rare. After methionine sulfoximine administration, glycogen as alpha and beta particles considerably increases. Gly: glycogen; magnification A and B: x 15000.

**Fig. (3):**
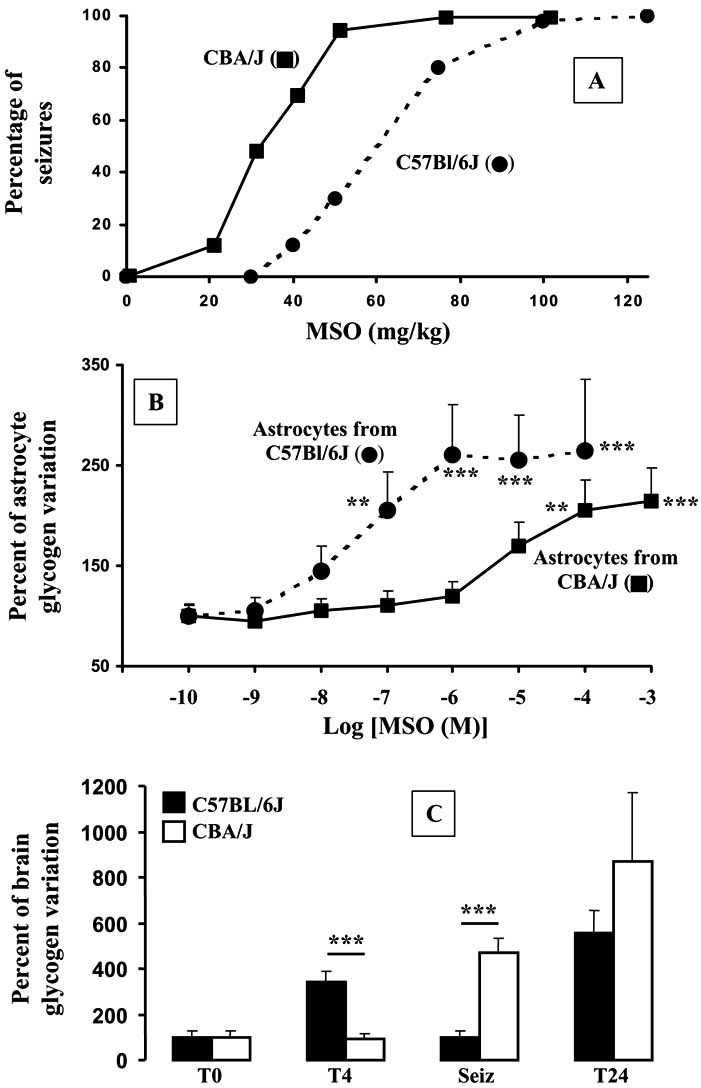
Effect of various doses of MSO, both *in vivo* (**A, C**) and *in vitro* (**B**) using two different strains of mice, C57BL/6J and CBA/J. **A**: Induction of seizures expressed as the percentage of convulsing mice as a function of MSO doses. **B**: Variation of *glycogen* determined in cultured astrocytes as a function of MSO doses. **C**: Variation of glycogen content of mice cortices sacrificed at either predetermined time T4 and T24) or at the onset of seizures (Seiz). Adapted from Bernard-Hélary *et al*. [151].

## ABBREVIATIONS

AMPA: = α-amino-3-hydroxy-5-méthyl-4 isoazole propionic acid
ANLSH: = Astrocytes-neuron lactate shuttle hypothesis
BBB: = Blood-brain barrier
CNS: = Central Nervous System
EEG: = Electroencephalography
FBPase: = Fructose-1,6-bisphosphatase
G-1-P: = Glucose-1-phosphate
G-6-P: = Glucose-6-phosphate
GABA: = γ-aminobutyric acid
GLAST: = Na^+^-glutamate co-transporter
Gln: = Glutamine
Glu: = Glutamate
GLUT: = Glucose transporter, facilitative type
GlycS: = Glycogen synthase
GP: = Glycogen phosphorylase
GS: = Glutamine synthetase
kDa: = kilo Dalton
Lac: = Lactate
LDH5: = Lactate dehydrogenase, type 5
MCT: = Monocarboxylate transporters
MSO: = Methionine sulfoximine
NMDA: = N-methyl-D-aspartate
Pyr: = Pyruvate
VIP: = Vasointestinal peptide
WHO: = World Health Organization
